# Outcomes of Osteosynthesis Versus Hemiarthroplasty in Elderly Patients With Arbeitsgemeinschaft Fur Osteosynthesefragen-Orthopedic Trauma Association (AO-OTA) 31A2 Hip Fractures

**DOI:** 10.7759/cureus.39795

**Published:** 2023-05-31

**Authors:** Nilesh Joshi, Sushil Mankar, Shantanu Deshkmukh, Vismay V Harkare

**Affiliations:** 1 Orthopaedics, N. K. P. Salve Institute of Medical Sciences & Research Centre and Lata Mangeshkar Hospital, Nagpur, IND

**Keywords:** osteosynthesis, harris hip score, ao/ota 31a2 femur fracture, intertrochanteric hip fractures, bipolar hemiarthroplasty

## Abstract

Background

Intertrochanteric femur fractures account for more than half of the cases of hip fractures. These injuries are among the most common fractures in elderly individuals. Elderly patients suffer from other comorbidities such as diabetes mellitus or hypertension and are prone to low surgical tolerance with increased postoperative morbidity and mortality. Although the ideal choice of treatment for intertrochanteric femur fractures in the elderly remains debatable, the use of hemiarthroplasty in elderly patients provides an early mobilization and decreased postoperative morbidity. In this study, we aimed to assess the functional outcomes of bipolar hemiarthroplasty and osteosynthesis in Arbeitsgemeinschaft Fur Osteosynthesefragen-Orthopedic Trauma Association (AO-OTA) 31A2 hip fractures using the Harris Hip Score.

Methodology

A total of 60 elderly patients with AO/OTA 31A2 hip fractures were divided into two groups and treated with bipolar hemiarthroplasty and osteosynthesis using proximal femoral nail (PFN). Functional scores were assessed at two months, four months, and six months postoperatively using the Harris Hip Score.

Results

The mean age of the patients was 73.03 ± 7.57 years in the study. The majority of the patients were females, 38 (63.33%), with 18 females in the osteosynthesis group and 20 females in the hemiarthroplasty group. The average operative time was 144.93 ± 9.76 minutes in the hemiarthroplasty group and 86.07 ± 11 minutes in the osteosynthesis group. Blood loss was 263.67 ± 42.95 mL in the hemiarthroplasty group and 84.5 ± 15.05 mL in the osteosynthesis group. The average Harris Hip Score at two months, four months, and six months was 64.77 ± 4.33, 72.67 ± 3.54, and 79.72 ± 2.53, respectively, for the hemiarthroplasty group and 57.83 ± 2.83, 64.13 ± 3.89, and 72.83± 3.89, respectively, for the osteosynthesis group (p < 0.001 for all follow-up scores). One death was encountered in the hemiarthroplasty group. Other complications included superficial infection noted in two (6.67%) patients in both groups. There was one episode of hip dislocation in the hemiarthroplasty group.

Conclusions

The use of bipolar hemiarthroplasty in intertrochanteric femur fractures in elderly patients can prove to be better in comparison to osteosynthesis, but the use of osteosynthesis can be effective for patients who cannot tolerate major blood loss and longer surgical times.

## Introduction

Intertrochanteric femur fractures are known to have the highest incidence of postoperative fatality among surgically treated fractures [1]. Involvement of the region of the proximal femur from the lesser trochanter before the beginning of the medullary canal up to the region of the neck outside the capsule is termed a per-trochanteric femur fracture [1]. Multiplanar stresses act upon these fractures postoperatively. The intersecting lamellar network which is tensile and compressive is injured due to fracture [1].

The incidence of hip fractures is reported to be 80 per 100,000 cases [2]. Intertrochanteric femur fractures comprise more than half of hip fracture cases [3]. Individuals aged more than 60 years form a major chunk of hip fracture patients.

Elderly patients usually suffer from other comorbidities such as diabetes mellitus and hypertension. Due to these associated conditions, these patients have low surgical tolerance which makes them prone to increased postoperative mortality due to prolonged bed rest and delayed mobilization [4]. Failure in reduction of the fracture, fractures with a basicervical pattern, communication in the posteromedial cortex, and fracture patterns that are reverse oblique are some of the factors that make an intertrochanteric femur fracture unstable [1]. The one-year mortality rate following a femur fracture of the per-trochanteric region is 14-36% [5].

Early mobilization with rapid recovery is the main aim of surgical management [5] to prevent complications such as pneumonia, pressure sores, osteoporosis, contractures, wasting of muscles, and deep vein thrombosis [6].

The best modality for the treatment of intertrochanteric femur fracture remains doubtful. Among the various treatment modalities available, such as external fixator application, proximal femoral nail (PFN), arthroplasty (partial or total), and screw plate system which has a dynamic hip screw, very few have proven to be efficient and appropriate for this kind of fracture pattern [2].

With the advent of the dynamic hip screw plate, it quickly became the preferred alternative for the management of intertrochanteric femur fractures. Association of Osteosynthesis/Association for the Study of internal fixation group in 1998 came out with the PFN which gained widespread popularity. Due to the decreased distance between the implant and the hip joint, the construct becomes more stable [7].

The failure rate seen with dynamic hip screws is about 6.8-9.8% and the PFN is 7.8-12.5% which is high in cases of unstable fractures [2]. Problems such as varus collapse, implant back-out, Z-effect, screw cut-out, and rotational instability are common postoperative complications [8]. A treatment modality that provides earlier mobilization and return to preoperative activity levels may be provided by both partial or total hip arthroplasty with little risk of mechanical failure [2].

This study aims to determine the outcome of fractures of the proximal femur with osteosynthesis and hemiarthroplasty.

## Materials and methods

This study was conducted in the Department of Orthopaedics, N.K.P. Salve Institute of Medical Sciences & Research Centre and Lata Mangeshkar Hospital, Nagpur. Approval was taken from the Institutional Ethics Committee (approval number: 59/2021). A total of 60 elderly patients with unstable intertrochanteric fractures of the femur were included, enrolled, and randomly allocated in groups using computer-based randomization software, with one group being treated with bipolar hemiarthroplasty and the other by osteosynthesis using PFN from December 2020 to October 2022. For classification and grading, Arbeitsgemeinschaft Fur Osteosynthesefragen (AO) classification was used. Patients with unstable intertrochanteric fractures of the femur (AO classification 31A2.2 and A2.3) and aged >65 years were included in the study. Follow-ups of all the patients were done in the second, fourth, and sixth months after surgery. Patients with fractures extending to the subtrochanteric region, aged <65 years, intertrochanteric fractures with ipsilateral lower limb fractures, those unfit for anesthesia and surgery, and those unwilling to undergo surgery were excluded from the study.

Preoperative evaluation

A detailed history of the patient was taken into consideration, including the mechanism of injury, associated injury, medical illness, and pre-injury status, along with a systemic and general examination to evaluate the complete status of the patient. Examination of the fractured limb, the status of the skin, and soft tissue was also performed. All routine preoperative hematological investigations were done for all patients. While the general condition was built up, the patient was optimized for surgery, skin traction was applied to the injured limb for temporary immobilization, and complications of recumbency were prevented as far as possible with good nursing care. Anesthesia fitness and physical fitness were noted.

A standard anteroposterior view of the pelvis with both hip joints and a lateral view of the affected hip were obtained in all cases to assess for fracture geometry and type.

Preoperative assessment and planning

Patients were kept nil by mouth for at least six hours before surgery. A xylocaine sensitivity test was done. Informed written consent was obtained from all patients. All patients in the study underwent routine preoperative preparation. Patients in the hemiarthroplasty group were administered intravenous cefuroxime 1.5 g 12 hours before surgery and subsequently one hour before incision along with 1 g of tranexamic acid.

Operative technique

Osteosynthesis

Patients were operated on under spinal or epidural anesthesia, as preferred and deemed necessary. Patients were placed on a fracture table in the supine position. An attempt at closed reduction was made in all cases and was checked in both the anteroposterior and lateral views. An incision was taken by palpating the tip of the greater trochanter and extending the incision proximally, and entry was taken with the help of bone awl at the tip of the greater trochanter. After removing the awl, a guide wire was inserted and an entry reamer was used to broaden the entry. Serial reaming was done to widen the femoral canal over the guide wire. A nail of adequate size was then pushed down until the proximal holes appeared to just pass the inferior aspect of the neck with the help of a jig. The guide wire was removed, 8 mm and 6.4 mm proximal drill holes were made, and appropriately sized bolts were placed. Two screws were placed in the available distal holes after drilling. Adequate wash was given and closure was done in layers after confirming the reduction under the image intensifier [9].

Hemiarthroplasty

Patients were operated on under spinal or epidural anesthesia, as deemed necessary. Patients were placed on the operation theater table in a lateral position with the affected side up. An incision was taken using a modified lateral (Hardinge) approach. The hip joint capsule was opened using an inverted T-shaped incision. After extraction of the femoral head and neck, a femoral canal was prepared with the help of a box chisel. The smallest size of the reamer was inserted first, and the reamer was directed toward the center of the femoral canal and sequential reaming was done. Femoral broaches were used next and broaching was started with at least two sizes lower than the anticipated stem size, and, subsequently, larger broaches were used. After placing the final broach, the femoral head size was determined by passing the extracted head through the head sizer. The trailhead and neck components were placed, the reduction was done, and the stability of the joint was assessed. The hip was dislocated again, and the trial components were removed. The femoral canal was obliterated using a cement restrictor and the femoral broach was passed again to ascertain the appropriate placement of the cement restrictor. Adequate wash was given in the femoral canal. The canal was dried and the polymethyl methacrylate bone cement was injected into the canal in a pressurized manner. The femoral component was inserted in the canal and held there with firm pressure. After the cement was hardened, an appropriately sized bipolar femoral head was fitted on the femoral stem. The hip joint was reduced and adequate wash was given. The closure was done in layers with a negative suction drain in situ [10].

In patients with uncemented hemiarthroplasty, after sequential reaming, the appropriate femoral stem was placed in the femoral canal, an adequately sized modular head was fitted on the stem, and then the hip was reduced.

Postoperative management

Intravenous (IV) antibiotics were given for two days postoperatively. Good analgesia was maintained and the limb was kept elevated. Patients were started on straight leg raising exercises, active knee mobilization, quadriceps strengthening exercises, and hip abductors strengthening exercises on day one for both groups. Active knee mobilization and full weight-bearing mobilization were started from postoperative day two for the hemiarthroplasty group. Sutures were removed on the 11th postoperative day if the wound was found to be healthy. Patients were followed up regularly clinically as well as radiologically as per pre-decided protocol.

Follow-up

**Figure 1:**
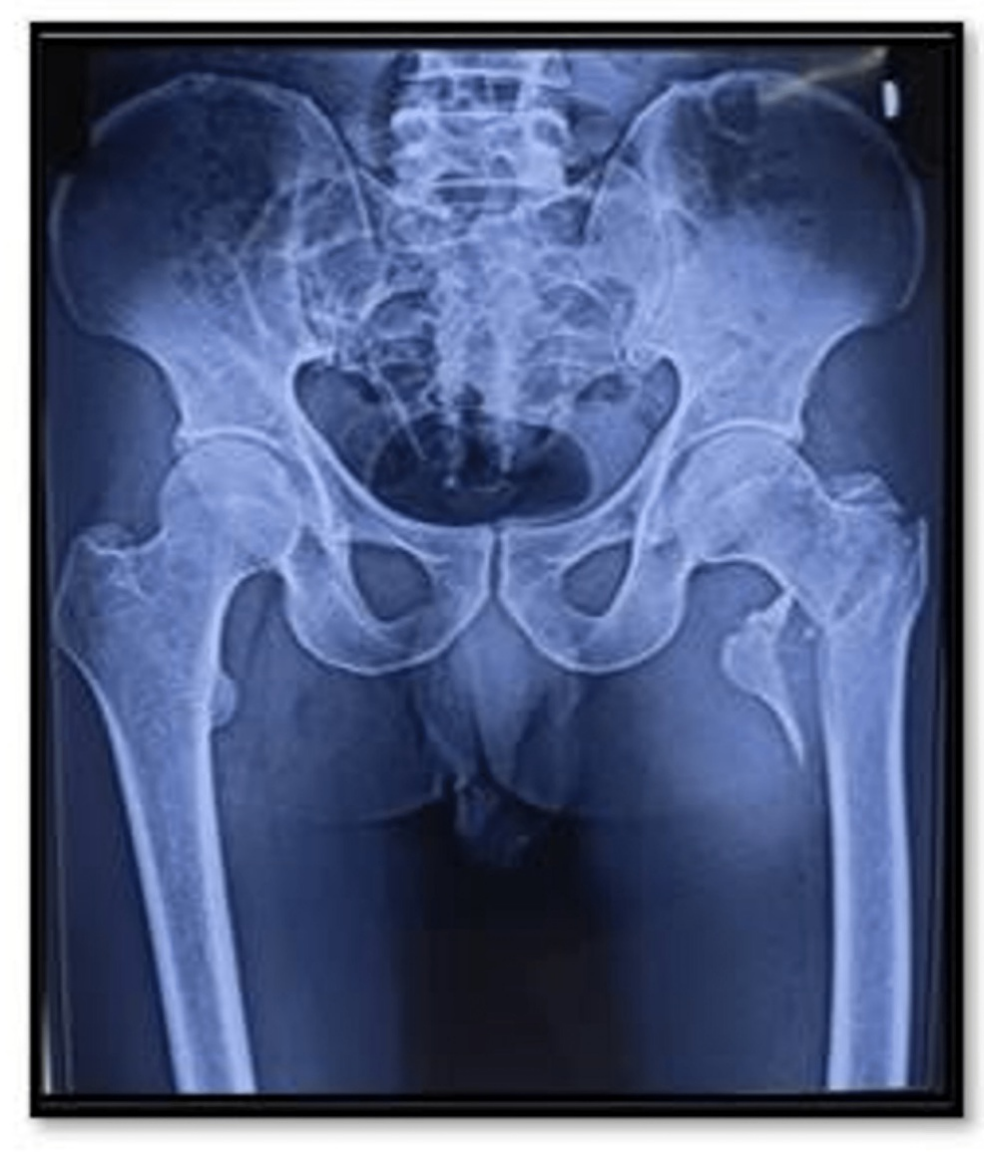
Preoperative X-ray of the osteosynthesis group.

**Figure 2:**
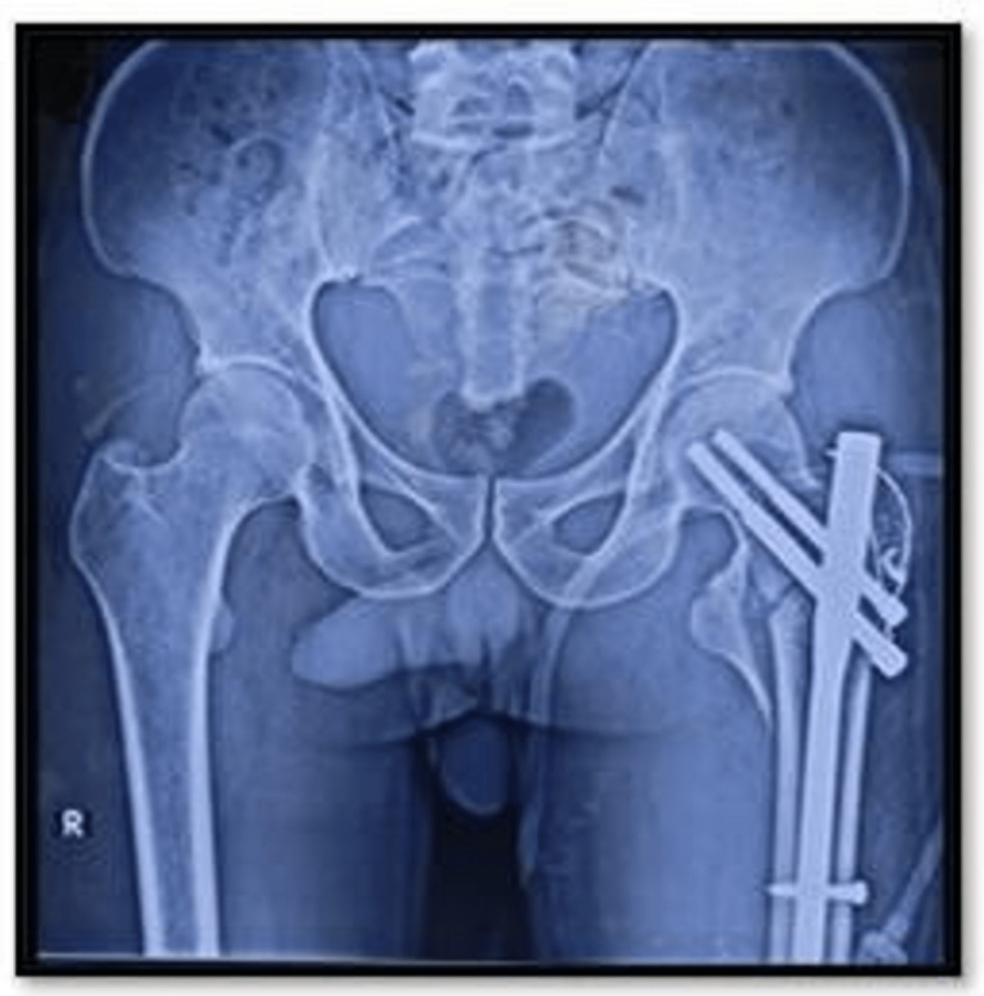
Postoperative X-ray of the osteosynthesis group.

**Figure 3:**
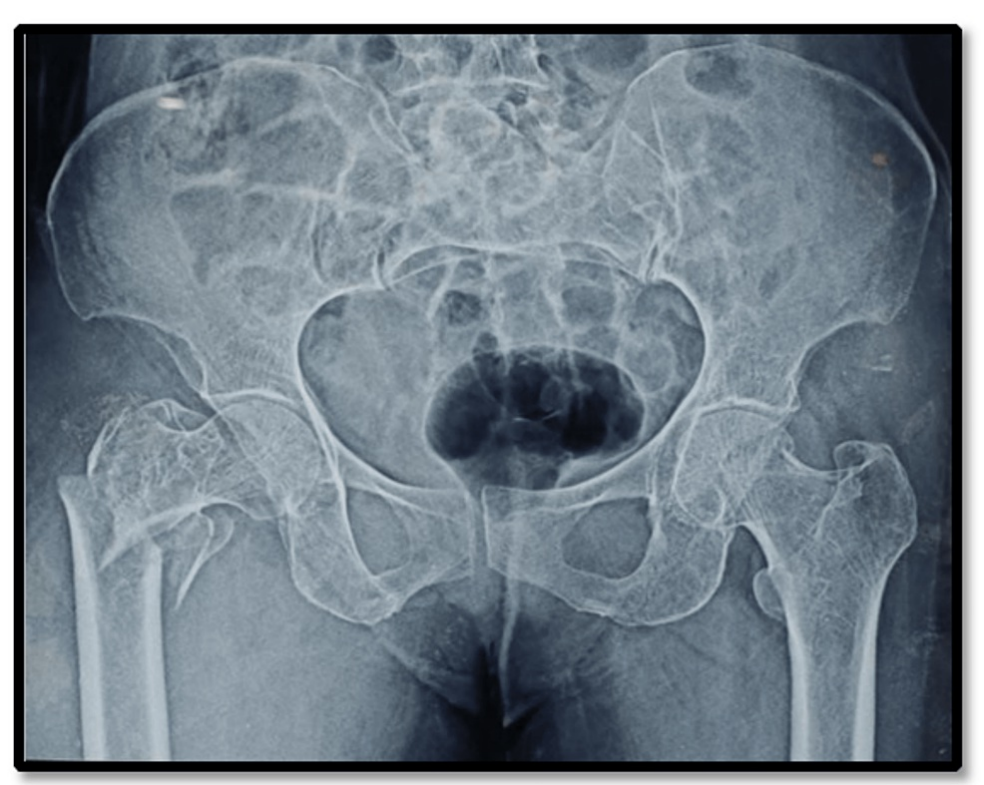
Preoperative X-ray of the hemiarthroplasty group.

**Figure 4:**
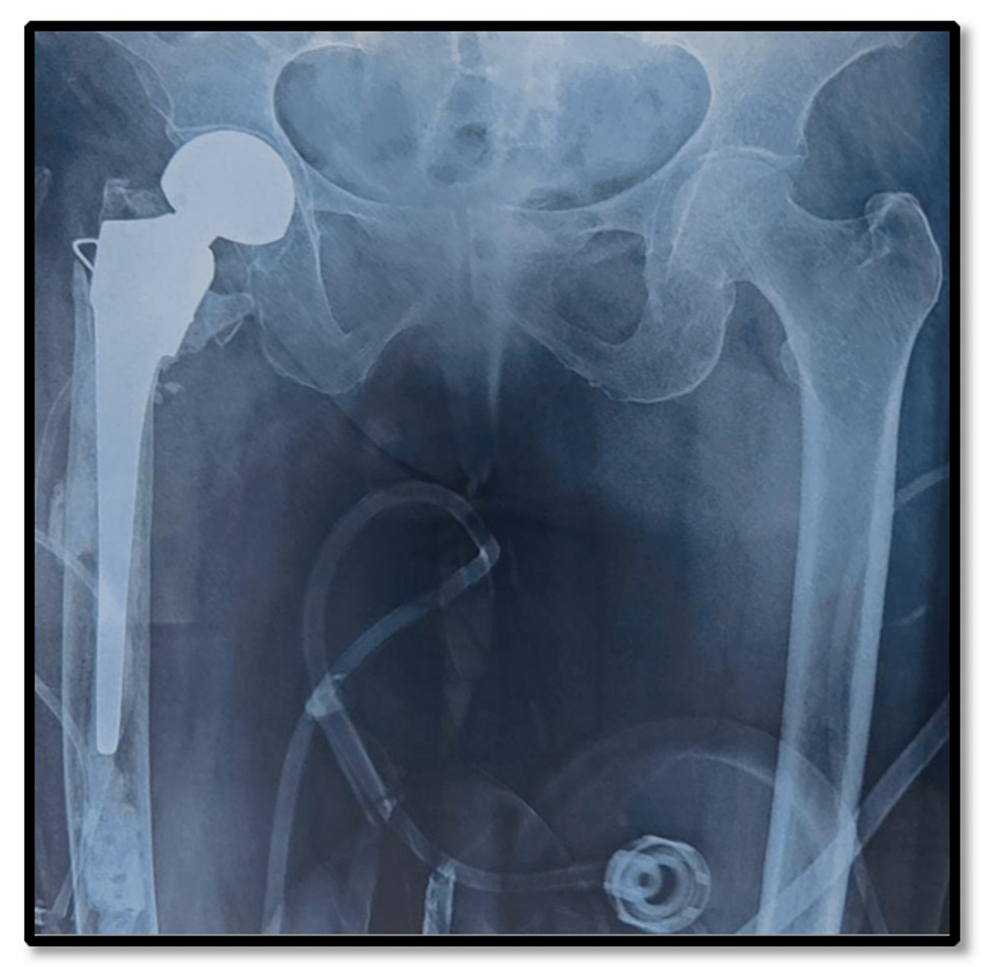
Postoperative X-ray of the hemiarthroplasty group.

The operated patients with unstable intertrochanteric femur fracture treated with bipolar hemiarthroplasty were followed postoperatively and assessed both clinically as well as radiologically regularly in the second month, fourth month, and sixth month. The functional outcomes were evaluated based on the Harris Hip Score. Preoperative and post-operative X-rays of the patients in both groups are shown in Figures 1-4.

Statistical analysis

At the end of the study, the data were analyzed statistically by the statistical program R. To determine the relationship, the chi-square test was used. A p-value <0.05 was considered significant.

## Results

The mean age of the study population was 78.03 ± 7.57 years, with the age ranging from 65 to 91 years. The average age in the hemiarthroplasty group was 73.13 ± 7.37 years and in the osteosynthesis group was 72.93 ± 7.9 years. The majority of patients were females, with 38 (63.33%) female and 22 (36.67%) male patients. The hemiarthroplasty group included 20 (66.67%) females and 10 (33.33%) males, whereas the osteosynthesis group included 18 (60%) females and 12 (40%) males. Among the study population, 55 (91.6%) patients had trivial trauma whereas five (8.4%) patients had a history of significant trauma (fall from height, road traffic accident).

The average blood loss in this study was 263.67 ± 42.95 mL in the hemiarthroplasty group and 84.5 ± 15.05 mL in the osteosynthesis group. The average operative time in the hemiarthroplasty group was 144.93 ± 9.67 minutes and in the osteosynthesis group was 86.07 ± 11 minutes. The average hospital stay was 12.7 days for both study groups (Table 1).

**Table 1:** Comparison of variables between the two groups.

Variables	Hemiarthroplasty group	Osteosynthesis group	P-value
Duration of surgery	144.93 ± 9.76 minutes	86.07 ± 11 minutes	<0.001
Hospital stay	12.83 ± 1.26 days	12.57 ± 1.33 days	0.4466
Blood loss	263.67 ± 42.95 mL	84.5 ± 15.05 mL	<0.001

Two patients in both surgical groups had an infection at the surgical site (6.67% each) and were managed with antibiotics. One of the surgical site infections was managed by debridement and adequate wash above the tensor fascia lata layer. No deep-seated infection was noted in this study. One episode of posterior dislocation of the hip was noted in the hemiarthroplasty group which was managed by closed reduction under general anesthesia and delayed weight-bearing. One incidence of death was seen in the hemiarthroplasty group after the fourth-month follow-up. Two patients in the osteosynthesis group had implant cut-outs (6.67%).

The average Harris Hip Score on the first follow-up was 64.77 ± 4.33 for the hemiarthroplasty group and 57.83 ± 2.83 for the osteosynthesis group. The scores at the fourth and sixth-month follow-ups were 72.67 ± 3.54 and 79.72 ± 2.53, respectively, for the hemiarthroplasty group and 64.13 ± 5.67 and 76.22 ± 4.77, respectively, for the osteosynthesis group. These scores were found to be highly significant (p <0.001) (Table 2).

**Table 2:** The Harris Hip scores at the follow-ups.

Variables	Hemiarthroplasty group	Osteosynthesis group	P-value
Harris Hip Score in the second month	64.77 ± 4.33	57.83 ± 2.83	<0.001
Harris Hip Score in the fourth month	72.67 ± 3.54	64.13 ± 3.89	<0.001
Harris Hip Score in the sixth month	79.72 ± 2.53	72.83 ± 3.89	<0.001

## Discussion

Eighty per 100,000 fracture cases are reported to be hip fractures [2]. More than half of hip fractures are intertrochanteric femur fractures [4]. With the elderly population increasing over the years, the majority of the patients in the emergency department are the elderly affected by some kind of trauma [1].

The utilization of treatment options such as external fixation, internal fixation using a screw plate system with a dynamic hip screw, PFNs, and partial or complete hip replacement, among others has advanced from the era of conservative care [2].

All current routine practices pose a significant risk of delayed return to pre-fracture activity level and increased risk of morbidity and mortality. Complications related to implant use such as varus collapse, implant back-out, Z-effect, screw cut-out, or rotational instability are the problems frequently faced by the use of PFNs [10].

The use of hemiarthroplasty or total arthroplasty helps against problems such as delayed mobilization of the patients and a decrease in the peri and postoperative morbidities such as thrombosis of deeper veins, pulmonary complications, and pressure sores.

The goal of this study performed in our tertiary care facility was to compare functional outcomes of hemiarthroplasty and osteosynthesis in patients with AO 31A2.2/2.3 hip fractures. The study involved 60 patients, with 30 participants in each group.

The average age in this study was 73.03 ± 7.57 years in both groups, with the average age being 73.13 ± 7.37 years in the hemiarthroplasty group and 72.93 ± 7.9 years in the osteosynthesis group. The results were similar to other studies reported in the literature. Gokay et al. [5] reported the average ages to be 76.2 years and 77.4 years in the hemiarthroplasty and osteosynthesis groups, respectively. Prasad et al. [2] reported similar results with ages of 67 years and 57 years in the hemiarthroplasty and osteosynthesis groups, respectively. Other studies by Dusak et al. [11] and Dash [12] also reported similar results. The distribution of the cases among gender was comparable in all the above-mentioned studies with predominance seen in the female population.

The surgical time in this study was 144.93 ± 9.76 minutes in the hemiarthroplasty group and 86.07 ± 11 minutes in the osteosynthesis group. There was a significant difference between the two (p < 0.001) (Table 3). Dusak et al. [11] and Patil [13] reported similar results.

**Table 3:** Comparison of surgical times among various studies.

Study	Hemiarthroplasty group (in minutes)	Osteosynthesis group (in minutes)
Nadir et al. 2105 [7]	52.33	28.19
Prasad et al. 2017 [2]	86	50
Dusak et al. 2019 [11]	180	85.63
Patil et al. 2008 [13]	110	102
Current study	144.93 ± 9.67	88.07 ± 11

Intraoperative blood loss was found to be significantly different among the two groups (p < 0.001), with the average blood loss in the hemiarthroplasty group being 263.67 ± 42.95 mL and 84.5 ± 15.05 mL in the osteosynthesis group (Table 4).

**Table 4:** Comparison of blood loss among various studies.

Study	Hemiarthroplasty group (in mL)	Osteosynthesis group (in mL)
Gokay et al. 2015 [5]	136.5	30.6
Prasad et al. 2017 [2]	310	100
Dusak et al. 2019 [11]	993	182.63
Venkatraman et al. 2019 [14]	300 ± 49.4	120 ± 29.4
Current study	263.67 ± 42.95	84.5 ± 15.05

The Harris Hip Score was calculated at the second, fourth, and sixth-month follow-ups and was found to be significant when compared between the two groups. The scores at the follow-ups for the hemiarthroplasty group were 64.77 ± 4.33, 72.67 ± 3.54, and 79.27 ± 2.53 for the hemiarthroplasty group and 57.83 ± 2.83, 64.13 ± 3.89, and 72.83 ± 3.89 for the osteosynthesis group at the second, fourth and sixth-month follow-up, respectively.

Saraf et al. [15] performed a similar study and found the results to be 61.4, 70.20, and 79.95 for the hemiarthroplasty group and 52, 64.05, and 79.65 for the osteosynthesis group at the second, fourth, and sixth-month follow-up. The results were similar to the present study.

Another study done by Vuppala et al. [6] reported similar results in which scores were calculated at the fourth and sixth-month follow-up, and significant results were found with scores being 80.55 and 83.25 in the hemiarthroplasty group and 68.89 and 72.47 in the osteosynthesis group, respectively. Venkatraman et al. [14] reported similar results in their study in favor of the hemiarthroplasty group.

The infection rate in our study was two in both groups (6.67%). The infection was superficial in nature in all the cases. No incidence of deep infection was noted in the study. The results of infection compared to other groups were similar, with Gokay et al. [5] reporting a rate of superficial infection to be 7.3% (five out of 68) for the hemiarthroplasty group and 5.33% (four out of 75) for the hemiarthroplasty group. They had no incidences of deep infection in the osteosynthesis group but two episodes of deep infection in the hemiarthroplasty group. Prasad et al. [2] also reported a similar result with four superficial infections in the hemiarthroplasty group (14.81%) and two episodes in the osteosynthesis group (7.4%). They also reported an incidence of deep infection in the hemiarthroplasty group (3.7%). Venkatraman et al. [14] reported two superficial infections in the hemiarthroplasty group (10%) and incidences were noticed in the osteosynthesis group.

There was one episode of dislocation in this study in the hemiarthroplasty group (3.33%). Other studies in the literature reported similar results, with Prasad et al. [2] reporting an incidence of 3.7% in the hemiarthroplasty group and Venkatraman et al. [14] reporting a single incidence of dislocation (5%) in the hemiarthroplasty group. While other authors such as Gokay et al. [5], Nadir et al. [7], and Patil [13] did not encounter an incidence of dislocation in their studies.

The incidence of conversion of hemiarthroplasty to total arthroplasty has been reported to be around 2.5% in a study with a two-year follow-up by Grosso et al. [16]. In a similar study with a mean follow-up of 5.1 years, a conversion rate of 7.3% was reported by Schmitz et al. [17]. No cases of conversion were noted in this study, but a higher follow-up is required for determining the conversion rate.

Study limitations

The study was conducted for a period of six months. A longer duration would provide better results. The sample size of our study was 30 patients in each group, a larger sample size would give a better result which could be more representative of the entire population. The Harris Hip Score is based on both subjective and objective parameters, and the score can be influenced by the subjective parameters which can depend on the willpower and the age of the patient. The study represents only one ethnicity which may have anatomical variations compared to the rest of the population.

## Conclusions

The use of bipolar hemiarthroplasty (cemented or uncemented) is an acceptable treatment modality for per-trochanteric femur fractures (AO 31A2 type). The Harris Hip Score for the hemiarthroplasty group was significant at the second, fourth, and sixth-month visits compared to the osteosynthesis group. The scores for the osteosynthesis group, though lesser at the initial follow-ups, were similar to the hemiarthroplasty group at the sixth-month follow-up. Hence, hemiarthroplasty allows for mobilization that can be performed early and early restoration to the pre-fracture activity level compared to osteosynthesis, which, in turn, reduces the postoperative morbidity and brings the patient to the pre-fracture activity levels at a faster rate. On long-term follow-up, both treatment modalities are similar in terms of the Harris Hip Score.

Certain drawbacks of hemiarthroplasty such as increased time of surgery and increased intraoperative blood loss were not seen in osteosynthesis, which hence could be a reason for preference of the surgical modality over hemiarthroplasty. Hemiarthroplasty can prove to be a better treatment modality for the treatment of AO/OTA 31A2-type hip fractures. The use of the PFN for osteosynthesis can be an effective surgery for patients who cannot sustain major blood loss and longer surgical times.
